# Rectus Femoris and Gastrocnemius EMG Driven Cheonjiin Speller for Korean Text Input

**DOI:** 10.3390/s25237243

**Published:** 2025-11-27

**Authors:** Ji Won Ahn, Gi Yeon Yu, Seong-Wan Kim, Young-Seek Seok, Seung Ho Choi

**Affiliations:** 1 Department of Biomedical Engineering, Yonsei University, Wonju 26493, Republic of Korea; jw010601@yonsei.ac.kr (J.W.A.); dbrldus074@yonsei.ac.kr (G.Y.Y.); 2 Department of Agricultural Biology, National Institute of Agricultural Sciences, Rural Development Administration, Wanju 55365, Republic of Korea; tarupa@korea.kr; 3 Gangwon-do Agricultural Product Registered Seed Station, Chuncheon 24410, Republic of Korea; 4 Department of Integrative Medicine, Major in Digital Healthcare, Yonsei University College of Medicine, Seoul 06229, Republic of Korea

**Keywords:** interface, rectus femoris, gastrocnemius, electromyography (EMG), speller system, Cheonjiin keyboard, directional input

## Abstract

Our study introduces a surface electromyography (sEMG)-based Cheonjiin speller system developed to assist individuals with restricted hand mobility. The interface incorporates a directional control framework—comprising up, down, left, right, and select commands—integrated with a Korean keyboard layout to enable efficient and accessible text input. Two-channel surface EMG signals were recorded from the rectus femoris and gastrocnemius muscles at a sampling rate of 200 Hz using an EMG acquisition module. The signals were processed in real time using notch and bandpass filtering, followed by full-wave rectification. To decode user intent, three physiologically interpretable time-domain features—root mean square (RMS), slope sign change (SSC), and peak amplitude—were extracted and subsequently used for classification. The Cheonjiin speller was implemented in Python 3.10.8 and operated through directional cursor navigation. System performance was quantitatively evaluated in two experiments: in Experiment 1, recognition accuracy for five discrete commands reached an average of 90.0%, while Experiment 2, involving continuous Korean word and sentence input, achieved an average accuracy of 88.65%. Across both experimental conditions, the system attained an average information transfer rate (ITR) of 96.19 bits/min, confirming efficient real-time communication capability. The results demonstrate that high recognition performance can be achieved using simple, low-computation features without deep learning models, confirming the feasibility of real-time implementation in resource-limited environments. Overall, the proposed speller system exhibits high operability, accessibility, and practical usability in constrained conditions and holds potential for integration into augmentative and alternative communication (AAC) systems for users with motor impairments. Moreover, its lightweight architecture, minimal computational load, and flexible directional control structure make it adaptable to a wide range of assistive and wearable technology applications.

## 1. Introduction

A Human–Machine Interface (HMI) provides a medium through which users communicate with computing systems, supporting the transmission of information and execution of commands. As computing systems become increasingly embedded in everyday environments, HMIs play a pivotal role in interpreting human intent and transforming it into precise machine control, thus determining the overall effectiveness of interactive technologies. Recent research has further expanded the concept of HMI to emphasize human–machine collaboration and mutual adaptation, highlighting the cognitive and behavioral mechanisms underlying user interaction with assistive systems [1]. This conceptual expansion has been reflected in the technological evolution of HMIs across different application domains.

Early developments in Human–Machine Interfaces (HMIs) were primarily centered on motor control applications, where bioelectrical or mechanical signals were converted into control commands for assistive devices such as robotic arms, prosthetic hands, and powered wheelchairs, which demonstrated that coordinated muscle synergies could be utilized to achieve stable control of multi-DOF systems [2]. These systems demonstrated the feasibility of translating physiological activity into reliable control outputs, marking an important milestone in the evolution of assistive HMI design [3,4,5,6]. Driven by these advances, digital and communication-oriented applications emerged, extending conventional control paradigms into more interactive and accessible environments. Virtual cursor control, on-screen joystick interfaces, and speller systems were developed to enable text input and menu selection using simplified input mechanisms, thereby providing alternative communication pathways for individuals with severe motor impairments. These developments marked a clear transition toward digital communication interfaces, where information exchange replaced direct physical control as the primary mode of interaction [7,8,9].

Among various input systems, the keyboard and mouse remain the most common means of user interaction. These devices achieve high efficiency by reliably translating hand movements into digital commands with both speed and accuracy. For users with unrestricted hand movement, they provide substantial convenience, as their operation is intuitive and requires no specialized training. Since keyboards and mouses were designed for users with free hand movement, difficulties may arise when used by those with limited hand movement [10]. To overcome these limitations, research continues on methods that allow users to input information or express intent without using hand movements. Consequently, interface technologies utilizing bio-signals are gaining attention as a new alternative.

Biomarker-based interface technology refers to the technology that interprets a user’s intent by receiving biological signals as input and controls devices or systems based on this interpretation [11]. Researchers have diversified their approaches toward bio-signal-based interaction, incorporating physiological and cognitive signals as intuitive communication channels. In this context, modalities such as electroencephalography (EEG), electrooculography (EOG), and electrocardiography (ECG) have emerged as complementary input channels [12,13,14].

Electrooculogram (EOG) is a signal generated by eye movements, representing corneal-retinal potential changes at levels of approximately 50 to 3500 μV [15]. Electroencephalogram (EEG) is an electrical signal generated by the cerebral cortex, typically ranging from approximately 0.5 to 100 μV [16]. Furthermore, Electromyogram (EMG) is an electrical signal generated during muscle contraction and relaxation, with a maximum signal amplitude reaching 50 μV to 20 mV [17].

Among these, EMG has the advantage of having a larger signal amplitude compared to other bio-signals and can be easily acquired from various body parts. Furthermore, EMG reflects muscle contraction intensity and patterns, enabling more accurate communication of user intent with simple movements. These characteristics make EMG a promising alternative to conventional communication devices such as keyboards and mouses, especially for individuals with limited hand mobility [18,19]. In particular, users with congenital limb deficiencies, amputations due to accidents, or impaired motor function due to disease can input characters using only simple muscle contractions through this structure. Because such users often lack the fine motor control required for conventional input devices, there is a growing need for systems that enable reliable communication through minimal muscle activity. To reduce the burden of complex hand movements, such systems often rely on simplified directional commands. By converting muscle contraction signals into digital inputs for four directions (up, down, left, right), characters can be selected in a more simplified manner without complex hand movements. Consequently, an EMG-based speller system with an up, down, left, right input structure can serve as a practical and highly accessible alternative for users with limited hand or arm movement.

To further enhance the practicality of such electromyography-based speller systems, it is essential to consider not only improvements in input and operation methods but also the selection of an efficient keyboard layout suited to the user’s conditions. Over the past decade, various EMG-based speller systems have been developed to explore muscle activity as an alternative communication channel. Early studies primarily focused on simple command recognition or single-letter selection tasks, demonstrating the feasibility of translating muscle contractions into discrete control inputs [20]. Subsequent research expanded these designs by integrating EMG with EEG or SSVEP signals to increase classification accuracy and target diversity [21]. However, most of these systems were optimized for English or alphanumeric input and relied on multi-channel setups or complex classifiers, limiting their accessibility and real-world usability.

Notably, few studies have examined language-specific optimization, and research on efficient Korean text input for individuals unable to use their upper limbs remains largely unexplored despite the language’s unique syllabic composition structure. Moreover, previous EMG-based speller studies have primarily utilized upper-limb or facial muscle signals, while no research has yet explored the feasibility of using lower-limb EMG—such as signals from the thigh or calf—for Korean text input. This indicates that prior work has not fully investigated the potential of muscle groups beyond the upper extremities, nor optimized speller architectures for languages with compositional structures like Hangul. To address these limitations, the present study investigates a low-channel, direction-based EMG speller employing the Cheonjiin (pronounced Chun-jee-in) keyboard layout, which enables efficient Hangul composition through minimal muscle activity in the lower limbs.

In Korea, text input is commonly performed using two main keyboard layouts, the QWERTY keyboard and the Cheonjiin keyboard. The widely used QWERTY keyboard layout has the advantage of enabling fast input based on the premise of free hand movement, as all consonants and vowels are assigned to individual keys. Although both layouts are optimized for users capable of precise finger control, individuals with limited motor function require an alternative input method that allows keyboard operation through a small number of simple EMG signals rather than fine hand movements. When the operation is restricted to limited directional inputs like up, down, left, and right, the QWERTY layout’s large number of keys and complex structure can lead to frequent key transitions during input, potentially making it inefficient. Therefore, in such limited input environments, it is necessary to identify a keyboard layout that minimizes unnecessary key movements and enables efficient character input through simple directional commands.

The Cheonjiin keyboard layout provides a promising solution for Hangul text entry under restricted input conditions. Based on the phonetic structure of the Korean language, which was systematically designed by King Sejong, each syllable is composed by combining basic consonant and vowel elements. The Cheonjiin keyboard, designed to efficiently implement this combinational principle, allows the input of all Korean consonants and vowels using only twelve keys—ten of which are dedicated to consonant and vowel composition—thereby reducing key movement and improving spatial efficiency. Unlike the QWERTY layout, where consonants and vowels are placed on separate keys that require frequent finger movements, the Cheonjiin layout enables users to compose both elements within a compact area through sequential directional inputs. For instance, when typing a syllable that includes the farthest consonant and vowel positions on a QWERTY keyboard, users may need to move across as many as eleven key intervals, whereas in the Cheonjiin layout, the same combination can be completed within only four directional inputs. This substantial reduction in key transitions shortens both physical and temporal distances during text entry, allowing users to efficiently generate characters through simple and logical directional sequences and thereby improving overall input efficiency.

This structural characteristic makes it suitable for electromyography-based speller systems, which select characters solely through up, down, left, and right inputs via muscle contraction. It enables high input efficiency even with limited input means. Meanwhile, realizing this input efficiency requires not only keyboard layout design but also signal processing and classification methods capable of effectively distinguishing electromyography signals.

Recent research in developing user interfaces utilizing electromyography (EMG) signals has primarily focused on applying complex preprocessing steps, including deep learning-based classification models and various feature combinations and normalization techniques. For example, this includes studies classifying hand gestures using CNN (Convolutional Neural Network)-based models [22] or employing LSTM (Long Short-Term Memory)-based neural networks to classify EMG signals into multiple grip gestures across different force levels in amputee subjects [23]. While these approaches offer high classification accuracy, they have the limitation of consuming significant computational resources [24]. In contrast, our study proposes a user interface capable of real-time input classification even in low-specification environments. This is achieved by using only simple time-domain features, such as Root Mean Square (RMS) and Slope Sign Changes (SSC), without complex neural network structures or high-dimensional feature combinations.

In our study, simple yet physiologically interpretable time-domain features were employed to enable real-time EMG classification under low-resource constraints. Specifically, features such as root mean square (RMS), slope sign changes (SSC), and peak amplitude were selected for their proven robustness and computational efficiency in representing lower-limb muscle activation patterns.

Our study introduces an EMG-based Cheonjiin speller system developed for individuals with restricted hand mobility. The interface adopts a four-directional control scheme (up, down, left, and right) using surface electromyography (sEMG) signals recorded from the rectus femoris muscle of the thigh and gastrocnemius muscle of the calf. Electrode placement and corresponding movement directions were determined according to anatomical considerations, and input commands were classified in real time by extracting time-domain features such as root mean square (RMS) and slope sign changes (SSC). Incorporating the Cheonjiin keyboard layout, which enables efficient directional control using a minimal number of EMG signals, allows efficient Korean text entry with fewer keystrokes compared to conventional methods. Compared to existing QWERTY-based input methods, the proposed system enhances higher accessibility and practicality for users with limited physical input capacity, thereby extending the applicability of EMG-based speller systems. Furthermore, through the adoption of a low-resource, real-time processing pipeline, the system minimizes computational load while maintaining high classification reliability. This simplicity makes the interface easily adaptable to portable or embedded assistive platforms.

This paper describes the design and implementation process of the proposed system and quantitatively evaluates its input recognition performance and actual Hangul input functionality through two experiments. The first experiment focuses on input action recognition accuracy, while the second centers on actual character input functionality. Results were analyzed based on metrics such as confusion matrix, accuracy, precision, recall, and F1-score. The proposed speller system can serve as an alternative input method for users who find conventional input devices difficult to use due to physical limitations. It also holds potential for future expansion into various assistive technology application environments, such as rehabilitation assistive devices and smart interfaces.

## 2. Methods

### 2.1. Participants

Three healthy participants (one male and two females, aged 22 to 28 years) were recruited to perform the Rectus Femoris and Gastrocnemius EMG driven Cheonjiin speller task. Participants were required to have no history of neurological disorders or movement impairment and the ability to communicate in Korean. The exclusion criteria included any conditions that could interfere with the experiment. Each participant reviewed and signed an informed consent form and was informed of their right to withdraw at any time without consequence. All personal information was anonymized to ensure confidentiality. All participants had prior experience with the Cheonjiin keyboard layout, which is widely adopted as the standard input method for everyday text entry on Korean mobile devices. Our study was approved by the Ethics Committee of the Yonsei University Wonju Institutional Review Board. (1041849-202411-BM-241-02).

### 2.2. Experimental Design

#### 2.2.1. Experimental Setup

The experiment was conducted in a quiet indoor environment free from electronic interference, with light and other environmental conditions kept constant to ensure signal quality and maintain participant concentration. Participants were seated on a comfortable chair with their legs relaxed, and the attachment status of the surface electromyography (sEMG) electrodes was verified before and after the experiment to ensure recording consistency.

The Cheonjiin keyboard was displayed on the monitor, where cursor movements and character input results were presented in real time to provide immediate feedback. Participants were instructed to operate the system by performing predefined voluntary contractions of the rectus femoris and gastrocnemius throughout the experiment. All inputs were performed spontaneously without external prompts, and the timing of each action was determined autonomously by the participants.

#### 2.2.2. System Design

**Figure 1:**
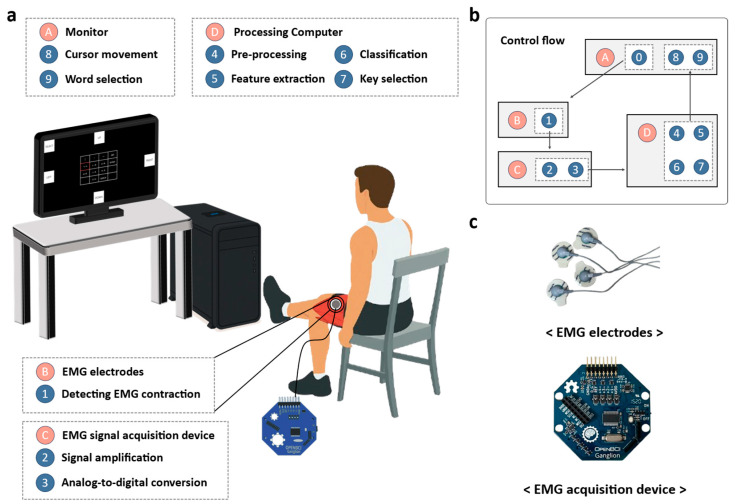
(**a**) System configuration showing EMG electrodes, an acquisition device, a PC running signal processing, and a monitor with the Cheonjiin keyboard; (**b**) Flow diagram of EMG detection, preprocessing, feature extraction, classification, and key selection; (**c**) Disposable Ag/AgCl electrodes (Kendall) and the Ganglion board (OpenBCI) used for EMG acquisition.

We developed an EMG-based Cheonjiin speller system that decodes four-channel EMG signals, with two channels obtained from the rectus femoris and two channels from the gastrocnemius muscles. Based on these signals, the system generated five distinct commands: four directional inputs (UP, DOWN, LEFT, RIGHT) and a selection (SELECT). The system comprises disposable Ag/AgCl electrodes for sEMG acquisition, an OpenBCI Ganglion board for analog amplification and 24-bit digitization, a computer platform for real-time signal processing and command classification. Visual feedback was simultaneously provided on a monitor through a graphical user interface, allowing users to confirm their inputs. Figure 1 presents a schematic overview of the system architecture and operational flow, illustrating the process from sEMG acquisition to feature extraction, classification, and visual feedback.

#### 2.2.3. Monitor Design

**Figure 2:**
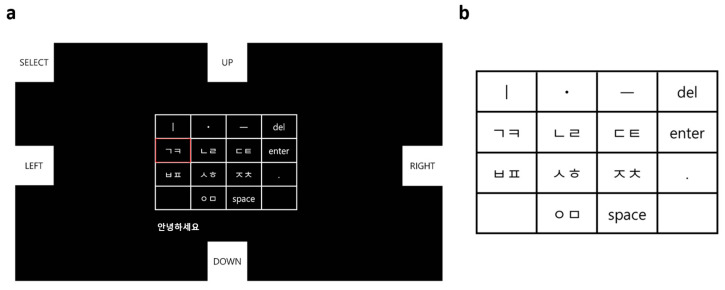
Monitor interface and keyboard layout of the EMG-based Cheonjiin speller. (**a**) The monitor interface displays directional command keys (UP, DOWN, LEFT, RIGHT, SELECT), which control cursor navigation and briefly flash when activated. The Cheonjiin keyboard layout is centered on the screen, and the text shown below it (e.g., “안녕하세요”) represents the user’s real-time input. The red box highlights the currently selected key; (**b**) The Cheonjiin keyboard layout consists of Korean vowels (ㆍ, ㅡ, and ㅣ) and grouped consonant keys (ㄱㅋ, ㄴㄹ, ㄷㅌ, ㅂㅍ, ㅅㅎ, ㅈㅊ, ㅇㅁ) arranged to reflect the principles of the Cheonjiin input system.

The EMG-based Cheonjiin speller system was implemented in a Python environment using the PyQtGraph and Pygame libraries. As illustrated in Figure 2, the virtual keyboard design consists of the Cheonjiin keyboard area and the character output area. A 4-row by 4-column grid-type keyboard is positioned centrally, containing a total of 14 input keys. In the top row, the keys ‘ㅣ’, ‘ㆍ’, and ‘ㅡ’ for vowel combinations are placed alongside the ‘del’ key. The lower rows include consonant input keys as well as function keys such as ‘space’, ‘enter’, and ‘.’. The key under the selection is highlighted with a red border to indicate its status.

To provide real-time visual feedback of the detected command, the color of the corresponding tile temporarily changed whenever an EMG-based directional command (UP, DOWN, LEFT, RIGHT, or SELECT) was recognized. This visual response allowed participants to confirm that each directional or selection command was successfully identified by the system before proceeding to the next action.

#### 2.2.4. Cheonjiin Keyboard Layout

The Cheonjiin (천지인) keyboard layout, derived from the Korean philosophy of heaven (ㆍ), earth (ㅡ), and human (ㅣ), organizes vowels and consonants based on the combination of these three fundamental elements. It was commercialized by LG Electronics in 2000 for mobile text input, and similar keypad structures have since been registered in several Korean patents [25]. The layout follows a 4 × 4 grid design containing 14 active keys, including consonant and vowel keys as well as functional keys such as space, enter, and delete. The vowel keys (ㆍ, ㅡ, ㅣ) are positioned in the top row, while the remaining rows contain grouped consonant and function keys. These elements form 21 vowels through single or combined selections, while consonants are arranged in grouped keys. The consonant and vowel composition rules of the Cheonjiin keyboard layout are summarized in Table 1.

Each consonant key includes multiple phonetic options that can be accessed through repeated selections. For instance, selecting the ‘ㄱㅋ’ key once produces ㄱ, twice ㅋ, and three times ㄲ. This mechanism also applies to other consonant keys such as ‘ㄷㅌ’, ‘ㅂㅍ’, and ‘ㅅㅎ’.

Vowel input is generated by combining one or more of the three vowel keys (ㆍ, ㅡ, ㅣ). For example, ㅏ is formed by combining ‘ㅣ’ and ‘ㆍ’, ㅗ by combining ‘ㆍ’ and ‘ㅡ’, and ㅟ by combining ‘ㆍ’, ‘ㅡ’, and ‘ㅣ’.

Once a complete syllable is formed by sequentially combining an initial consonant (choseong), a medial vowel (jungseong), and, when applicable, a final consonant (jongseong), the enter key is used to confirm the input. Spacing between words is created using the space key, allowing continuous text generation in Korean. This compact structure enables efficient character composition using minimal key operations—an essential feature for assistive or EMG-based input systems with limited command bandwidth.

### 2.3. Signal Acquisition and Preprocessing

#### 2.3.1. Signal Acquisition

For our study, two pairs of lower-limb muscles—the left and right rectus femoris and the left and right gastrocnemius—were targeted. The rectus femoris, a major component of the quadriceps group, primarily contributes to knee extension and hip flexion during voluntary leg movements, while the gastrocnemius, a superficial calf muscle, is responsible for ankle plantarflexion and contributes to postural stability. These muscles were selected not only for their anatomical accessibility but also for their substantial size and functional independence, which enable distinct activation patterns with minimal crosstalk between limbs. Due to their size and superficial fiber orientation, both muscles generate strong surface EMG signals during contraction, facilitating stable and high-quality signal acquisition. Moreover, their robust contractile properties allow consistent performance across repeated activations, making them suitable for long-duration control tasks in EMG-based interfaces.

EMG signals were acquired using a single active electrode for each target muscle, with a common reference electrode placed on the patella to minimize electrical interference. The active electrodes were aligned parallel to the muscle fiber orientation to maximize the detected potential amplitude and reduce phase effects, and a minimum distance of 3 cm was maintained between the active and reference electrodes to prevent signal interference and minimize the influence of shared electrical fields. For the rectus femoris muscles, electrodes were attached bilaterally to the central portion of each muscle belly, approximately midway between the anterior superior iliac spine and the patella, whereas for the gastrocnemius muscles, electrodes were placed on the most prominent region of the medial belly on both legs. This bilateral configuration allowed symmetrical signal acquisition while minimizing crosstalk between adjacent muscle groups.

**Figure 3:**
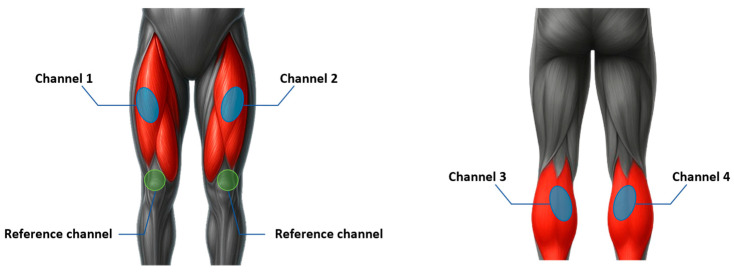
Electrode placement for EMG signal acquisition from the rectus femoris and gastrocnemius.

Accurate electrode positioning is essential for maintaining high signal quality and classification accuracy, as misalignment or inconsistent spacing can result in amplitude attenuation, phase distortion, or motion artifacts. Figure 3 illustrates the anatomical placement of the EMG sensors on the bilateral rectus femoris and gastrocnemius muscles.

The EMG signals were measured using a disposable Ag/AgCl electrode (Kendall, H135SG, Cardinal Health, Mansfield, MA, USA) with dimensions of 43 mm × 35 mm. The signals were transmitted via button-type cables to the EMG signal acquisition device (OpenBCI, Ganglion board, OpenBCI Inc., Brooklyn, NY, USA), which incorporates an analog front end (MCP3912) with built-in amplification and 24-bit A/D conversion [26,27]. This hardware configuration provided a robust and scalable platform for integrating EMG signals.

#### 2.3.2. Signal Preprocessing

**Figure 4:**
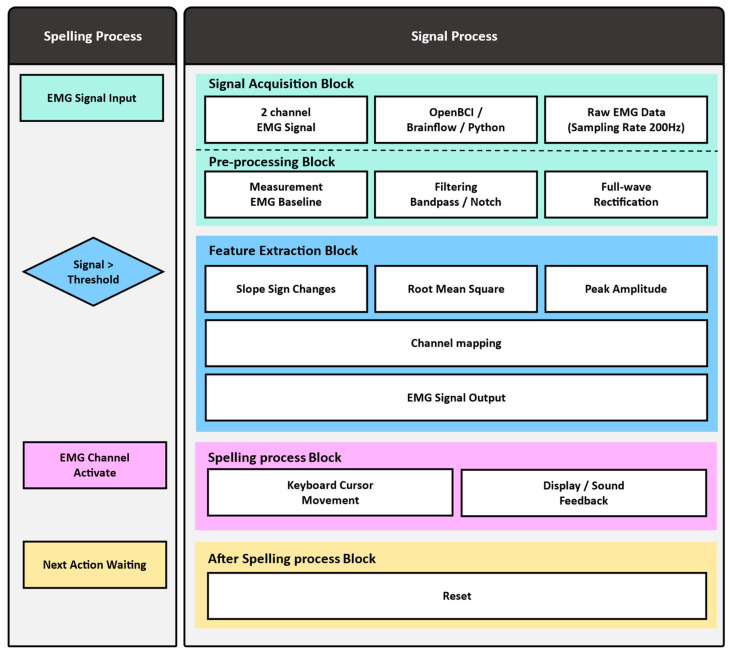
Flowchart of the proposed EMG-based Cheonjiin speller system, showing signal preprocessing and cursor control steps.

Figure 4 schematically illustrates the signal processing and cursor control flow of the directional key-based Cheonjiin speller system using electromyography (EMG). In our study, a total of four channels of EMG signals were collected in real time using an EMG signal acquisition device via electrodes attached to the bilateral rectus femoris and gastrocnemius muscles. The analog signals were amplified and digitized at 200 Hz using the OpenBCI Ganglion board and transmitted to Python through the BrainFlow library. To ensure reliable control performance, the acquired signals were subjected to sequential preprocessing and feature extraction steps.

EMG preprocessing was implemented in Python using standard IIR filtering routines to ensure real-time compatibility with the OpenBCI Ganglion board. Raw EMG data from each channel were first passed through cascaded notch and bandpass filters designed to attenuate power-line interference and retain physiologically relevant frequency components, while complex deep learning based recognition approaches such as LSTM or wavelet–hybrid models have also been proposed in related EEG and EMG research [12]. Specifically, second-order IIR notch filters were applied sequentially at 50 Hz and 60 Hz (quality factor Q = 30) to eliminate regional powerline and hardware-induced noise. Subsequently, a fourth-order Butterworth bandpass filter (20–90 Hz) was employed to preserve the dominant EMG spectrum while suppressing motion artifacts and high-frequency noise beyond the motor unit firing range.

After filtering, the signals were processed by full-wave rectification to obtain an absolute-valued envelope suitable for amplitude-based feature extraction. A sliding analysis window of 1.0 s with a 5 ms updated step was applied for continuous computation of time-domain features. Within each window, root-mean-square (RMS) and slope-sign-change (SSC) values were extracted to represent muscle activation intensity and transition frequency, respectively. The window length was empirically optimized to balance temporal resolution and computational efficiency under the low-resource constraints of the Ganglion board, ensuring reliable real-time operation (<10 ms latency per iteration). These preprocessing steps collectively provided stable, artifact-suppressed EMG signals suitable for accurate directional command classification [28], emphasizing the importance of stable sEMG morphology and fatigue-resistant signal characteristics in long-term use. The processing pipeline was designed to operate with minimal computational delay, ensuring compatibility with real-time EMG-based control requirements.

#### 2.3.3. Feature Extraction

Time-domain features were selected for their proven reliability in EMG-based classification and suitability for real-time implementation. Among various candidates, RMS, SSC, and peak amplitude were chosen based on their physiological interpretability and consistent discriminative performance reported in prior EMG studies. These features have been extensively employed in surface EMG analysis for their ability to capture distinct yet complementary aspects of neuromuscular activation [29,30]. Specifically, RMS quantifies the effective power of the EMG signal and correlates with the level of motor unit recruitment, making it a reliable indicator of contraction strength. SSC measures the frequency of slope reversals between successive samples, providing sensitivity to transient changes in contraction dynamics and muscle fatigue. Peak amplitude serves as an auxiliary descriptor that reflects short-duration, high-intensity activations which may not be fully represented by RMS alone.

Although alternative time-domain features such as zero crossing (ZC) and waveform length (WL) were also considered, they were excluded after empirical evaluation due to their high sensitivity to baseline noise and microfluctuations, which often caused instability in short-window or high-amplitude EMG recordings. Furthermore, ZC becomes ineffective after full-wave rectification because polarity transitions are no longer observable [31]. Given that lower-limb EMG signals in our study exhibited strong amplitudes and clear separability across directional commands, the inclusion of additional or noise-prone features would not have improved classification performance but would have increased computational latency.

Because of these characteristics, the system did not require complex classification algorithms or normalization procedures. Instead, a simple, threshold-based decision rule was adopted, enabling intuitive operation and ensuring low computational load suitable for real-time embedded EMG control. Collectively, the selected features—RMS, SSC, and peak amplitude—provide physiologically interpretable and computationally efficient representations of muscle activity, ensuring reliable and low-latency operation for real-time EMG-based control [32], contrasting with recent studies that apply high-dimensional sEMG features for diagnostic classification.

The first feature, the root mean square (RMS), quantifies the energy content of the signal within a given analysis window and is defined as

(1) $$RMS=\sqrt{\frac{1}{N}\;\sum_{i=1}^{N}x_{i}^{2}}$$

where $x^{i}$ denotes the i-th sample of the rectified signal and N is the number of samples in the window.

The second feature, slope sign change (SSC), captures the frequency of slope transitions between consecutive samples, serving as an indicator of contraction irregularity. It is computed as

(2) $$SSC=\sum_{i=2}^{N-1}f[{(x}_{i}-x_{i-1})\times {(x}_{i}-x_{i+1})]$$

with the condition function

(3) $$f\left(x\right)=\left\{\begin{array}{c} \;1,\;\;\;\;if\;\;x>threshold\\ 0,\;\;\;\;otherwise\;\;\;\;\;\;\;\;\;\;\;\;\; \end{array}\;\right.$$

where $x^{i-1}$, $x^{i}$, $x^{i+1}$ represent three consecutive samples. The threshold is introduced to reduce false detections caused by minor fluctuations due to noise. A higher SSC value indicates more frequent slope changes, implying greater variability in muscle activation.

The third feature, the maximum amplitude, was included as an auxiliary indicator to capture the peak intensity of muscle contraction within each analysis window. While RMS represents the overall energy and SSC reflects signal irregularity, the maximum amplitude emphasizes transient high-magnitude activations. Incorporating this feature improved the robustness of channel activation detection by reducing misclassifications due to weak or noisy contractions.

By jointly evaluating RMS, SSC, and maximum amplitude in real time, the system determined whether each channel was active, which provides a simple yet effective control logic compared to neural learning–based approaches for muscle-driven actuation [33]. The resulting channel activations were then translated into directional cursor movements according to the predefined command mapping rules.

**Figure 5:**
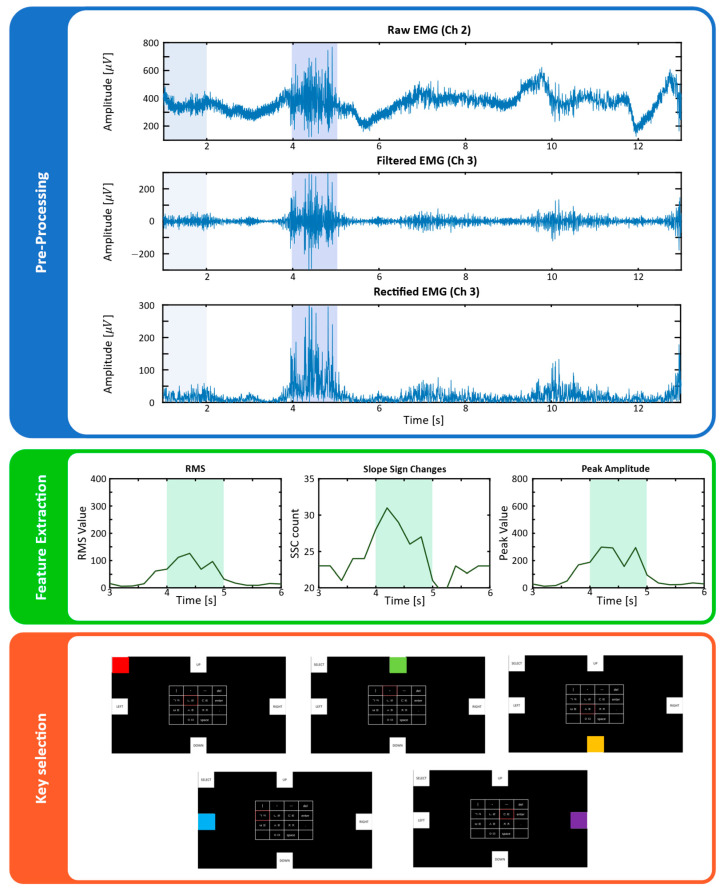
Signal processing and feature extraction of EMG signals, including preprocessing, feature computation (RMS, slope sign changes, and peak amplitude), and key selection. In the preprocessing plots, the light blue region (0~2 s) represents the baseline measurement period, whereas the darker blue region indicates the segment used for signal processing. The light green region shown in feature extraction plots corresponds to the same processing segment highlighted in the prepro-cessing stage.

As shown in Figure 5, the system first measures and stores the baseline RMS value for each channel during the initial stable state. It then compares this baseline with the signal received in real time to determine channel activation. A channel was deemed active if its RMS value increased significantly above the baseline and simultaneously exceeded predefined thresholds for SSC and peak amplitude.

#### 2.3.4. Classification

The extracted time-domain features (RMS, SSC, and peak amplitude) were compared with baseline values to determine whether each channel was active. A channel was deemed active if its RMS increased significantly above the baseline while simultaneously exceeding the thresholds for SSC and peak amplitude.

**Figure 6:**
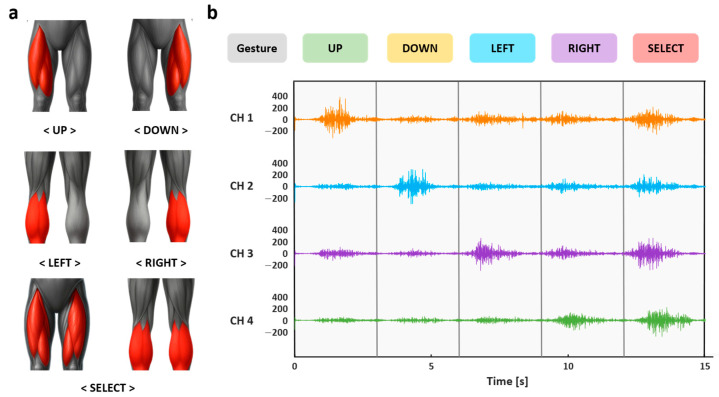
(**a**) Illustration of five input gestures based on rectus femoris and gastrocnemius muscle activation; (**b**) EMG signal recordings from individual channels showing variations during muscle contraction and relaxation for each gesture.

Based on this classification, user actions were mapped to five distinct control commands. As illustrated in Figure 6, contraction of channel 1 was interpreted as an UP command, while contraction of channel 2 was interpreted as a DOWN command. Similarly, contraction of channel 3 corresponded to a LEFT command, and contraction of channel 4 corresponded to a RIGHT command. Finally, simultaneous contractions of two or more channels sustained for more than 0.3 s were recognized as a SELECT command.

This mapping strategy maximized the separability of commands while minimizing cognitive and physical load, allowing participants to reliably control four directional cursor movements and confirm selections in real time. The classification results were immediately reflected in the graphical user interface, enabling efficient cursor control and character input.

### 2.4. Experimental Procedure

Two experiments were conducted to evaluate the feasibility and usability of the proposed EMG-based Cheonjiin speller system. The first experiment, basic command recognition evaluation, examined the recognition performance of EMG-based directional commands, while the second experiment, the real-time Korean text input evaluation, evaluated the system’s usability during continuous text-entry tasks.

The basic command recognition evaluation (Experiment 1) focused on verifying the system’s ability to accurately recognize and classify EMG-based directional commands, while the real-time Korean text input evaluation (Experiment 2) aimed to assess its usability under continuous operation in a realistic text-entry environment. These two experiments provided a complementary assessment of the system’s overall performance, linking recognition accuracy and information transfer rate (ITR) to practical text-input functionality.

**Figure 7:**
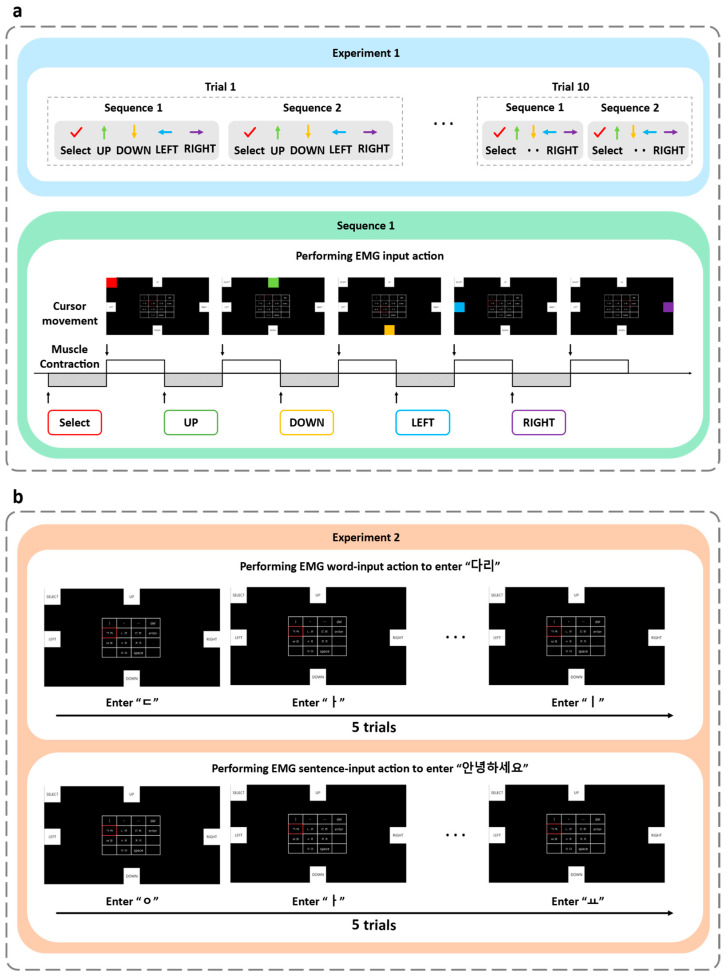
(**a**) Experiment 1 illustrating the control of five distinct commands (UP, DOWN, LEFT, RIGHT, and SELECT) through EMG gestures; (**b**) Experiment 2 illustrating the word-input task and sentence-input task using EMG-based directional control.

Participants were seated at 70 cm from the monitor and instructed to perform rectus femoris and gastrocnemius contractions to control cursor movement, enabling the selection of consonants and vowels on the virtual keyboard. Two experiments were designed using guided target selection tasks. These experiments were conducted to evaluate the system’s gesture recognition performance and Hangul input functionality. Figure 7 illustrates the experimental procedure.

#### 2.4.1. Basic Command Recognition Evaluation—Experiment 1

Experiment 1 was conducted to verify the feasibility of the proposed EMG-based Cheonjiin speller system by evaluating its ability to accurately recognize muscle-induced directional commands—UP, DOWN, LEFT, RIGHT, and SELECT. This experiment aimed to verify the system’s ability to consistently recognize each command on a per-key basis, thereby establishing the reliability of its fundamental recognition mechanism prior to real-time operation.

Based on two fundamental muscle gestures, participants controlled five different commands through single, repeated, or simultaneous contractions. Each of the five commands was performed twice, resulting in 10 key operations per set, and the sequence was repeated for a total of ten trials, as shown in Figure 7a. During each action, the system’s real-time recognition accuracy was verified and recorded. In each trial, participants executed a single command, then rested for 2 s before performing the next command. A total of 10 trials were conducted per with approximately 30 s of rest between successive trials.

#### 2.4.2. Real-Time Korean Text Input Evaluation—Experiment 2

Experiment 2 was designed to verify whether the proposed system could function as a practical Korean text-input interface by evaluating its usability through a continuous text-entry task. This experiment assessed the system’s capability for real-time control and continuous text typing, examining whether consecutive EMG-based commands could be reliably recognized and translated into continuous text control during actual input tasks.

To this end, participants were instructed to reproduce the word “다리” (meaning “leg”) by sequentially selecting the corresponding consonant and vowel characters using the EMG-controlled cursor. And this word-input task was repeated across five trials, with a 30 s rest interval between trials, as presented in Figure 7b.

Subsequently, participants performed the sentence-input task by reproducing “안녕하세요” (meaning “hello”) five times under the same conditions, with identical rest intervals between trials. During both tasks, participants sequentially selected consonants and vowels to compose characters and finalized each syllable using the ‘enter’ key. The system simultaneously recorded the recognition results for each input, enabling evaluation of recognition accuracy and overall usability of the proposed interface.

### 2.5. Data Analysis

To quantitatively analyze the results, we employed standard performance metrics derived from a confusion matrix, a widely used tool for evaluating the performance of classification algorithms by summarizing predicted and actual outcomes across all classes [34]. The confusion matrix consists of four fundamental elements based on True/False and Positive/Negative. When a user performs a specific input action and the system accurately recognizes it as that action, it is defined as a True Positive (TP). Conversely, when a user performs a specific input action, but the system incorrectly recognizes it as a different action, it is defined as a False Negative (FN). Conversely, when a user performs a different input action and the system correctly determines it is not that action, it is called a True Negative (TN). When a user performs a different input action, but the system incorrectly recognizes it as that action, it is called a False Positive (FP). Based on these values, precise performance evaluation metrics can be calculated. Our study used accuracy, precision, recall, and F1-score as the primary evaluation metrics [35].

Accuracy was defined as the ratio of correctly classified samples to the total number of samples:

(4) $$Accuracy=\frac{TP+TN}{TP+TN+FP+FN}\;\times \;100$$

Precision represents the proportion of predicted positives that were correct and was defined as:

(5) $$Precision=\frac{TP}{TP+FP}\;\times \;100$$

The recall rate, corresponding to the proportion of actual positives correctly identified, was expressed as:

(6) $$Recall=\frac{TP}{TP+FN}\;\times \;100$$

Finally, the F1-score, the harmonic mean of precision and recall, was calculated as:

(7) $$F_{1}score=\frac{2\cdot Precision\cdot Recall}{Precision+Recall}$$

The F1-score is widely used as a useful metric for evaluating the overall performance of classifiers, particularly in situations where class imbalance exists. In our study, these metrics were also utilized to quantitatively verify the motion recognition accuracy and character input stability of the proposed system.

In addition to these classification metrics, the information transfer rate (ITR) was calculated to quantitatively evaluate the communication efficiency of the proposed system. The ITR, expressed in bits per minute (bits/min), was computed using the standard formula widely adopted in BCI and EMG-based communication studies:

(8) $$ITR={[log}_{2}N+P{log}_{2}P+(1-P){log}_{2}(\frac{1-P}{N-1})]\times \frac{60}{T}$$

where N is the number of available commands (five in our study), P denotes the classification accuracy, and T represents the average time required for one selection (in seconds). This metric jointly considers both recognition accuracy and selection speed, thereby providing an integrated measure of system efficiency for real-time operation.

## 3. Results

### 3.1. Feature Analysis

**Figure 8:**
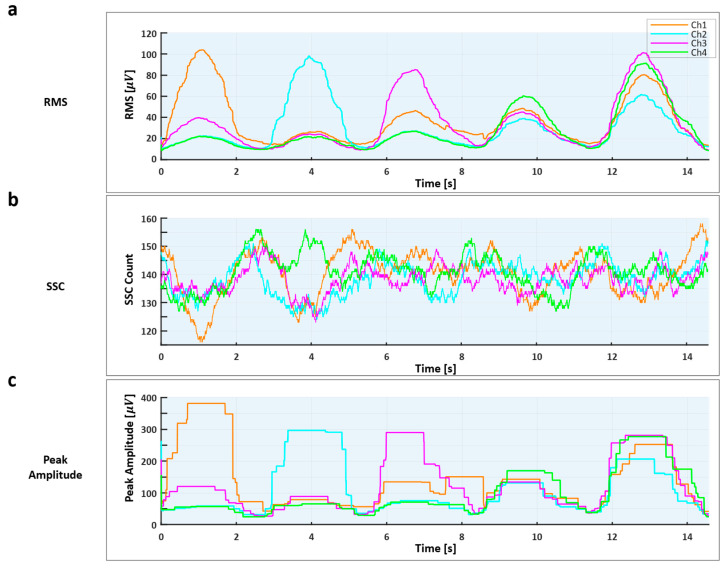
Temporal variations in the three extracted time-domain features during continuous directional input sequences. (**a**) Root Mean Square (RMS) representing overall signal power and muscle activation strength; (**b**) Slope Sign Change (SSC) capturing rapid fluctuations in motor-unit firing activity; (**c**) Peak Amplitude highlighting transient high-intensity contractions observed across four EMG channels.

As illustrated in Figure 8, the temporal patterns of the three extracted features—RMS, SSC, and maximum amplitude—clearly represent muscle activation and relaxation phases across channels during continuous directional input sequences. RMS exhibits distinct amplitude peaks corresponding to active contractions, SSC captures fine-grained variations in firing activity, and the maximum amplitude highlights transient bursts of high-intensity contraction. These complementary patterns enable robust detection of active channels through simultaneous thresholding of all three features. When visualized separately for Experiment 1 and Experiment 2, the distributions of these features showed consistent separability among the target commands (e.g., UP, DOWN, SELECT), confirming stable real-time classification during sequential text-entry tasks.

### 3.2. Experimental Results

Two experiments were conducted to quantitatively evaluate the performance of the EMG-based Cheonjiin keyboard system. In Experiment 1, participants were instructed to perform each of the five commands—up, down, left, right, and select—individually, allowing assessment of recognition accuracy for single inputs. In Experiment 2, participants were tasked with performing multiple commands consecutively to simulate actual character input, thereby enabling evaluation of the system’s stability and consistency under real-world usage conditions.

**Figure 9:**
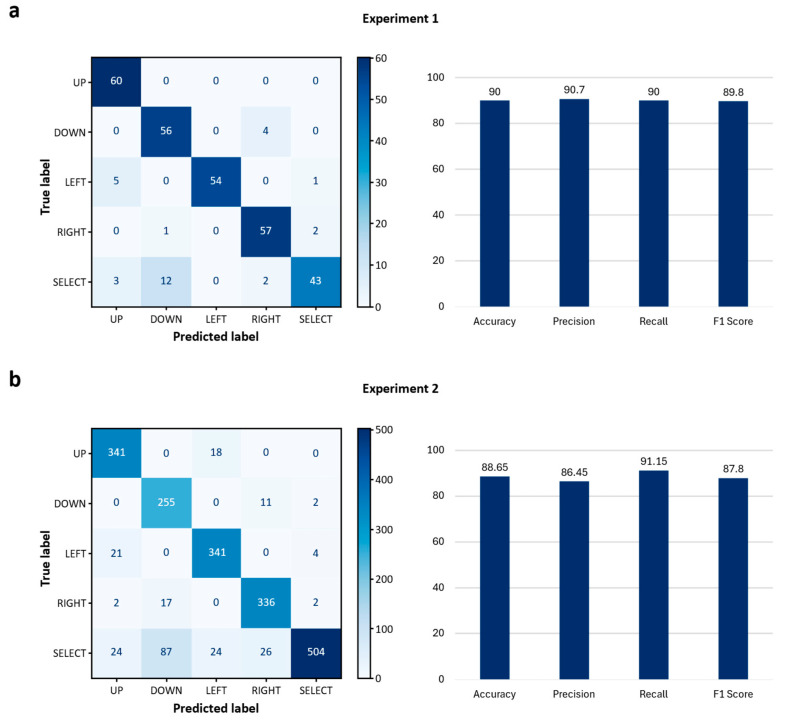
(**a**) Confusion matrix for Experiment 1; (**b**) Confusion matrix for Experiment 2, with corresponding bar graphs presenting the average accuracy, precision, recall, and F1-score values, expressed as percentages.

Experiment 1 was conducted with a total of 3 subjects, each of whom repeatedly performed the 5 input actions, which are ‘up’, ‘down’, ‘left’, ‘right’, and ‘select’. According to the confusion matrix derived from the collected 300 data points, the proposed system demonstrated high classification accuracy across most classes. As shown in Figure 9a, the system achieved perfect classification for the ‘up’ class, while the ‘left’, ‘right’, and ‘down’ classes also demonstrated high accuracy with only a few misclassifications. In contrast, the ‘select’ class exhibited the lowest accuracy, frequently confusing it with the ‘left’ and ‘up’ classes.

Based on these classification results, the overall average accuracy was 90.0%, precision was 90.7%, recall was 90.0%, and the F1-score was 89.8%. Notably, the ‘up’, ‘left’, and ‘right’ inputs demonstrated high recognition performance, with precision and recall values close to or above 90%. In contrast, the ‘select’ input showed the lowest recall at 71.7%, despite a relatively high precision of 93.5%, indicating that many ‘select’ actions were misclassified as other directional inputs. Analysis of the information transfer rate (ITR) showed that the average ITR for the three subjects was 99.5 bits/min, suggesting that the proposed system ensured sufficient speed for rapid recognition and processing of input actions.

The results of this experiment demonstrate that the system maintains high overall recognition performance despite differences in signal magnitude, duration, and variation patterns among subjects. Furthermore, it was confirmed that adequate classification performance can be achieved using only simple feature extraction algorithms based on SSC and RMS.

Experiment 2 involved collecting a total of 1704 data points by having three subjects repeatedly perform each input action. The multi-class classification performance of the proposed system was evaluated through a confusion matrix, as illustrated in Figure 9b. The results showed that the ‘up’, ‘down’, ‘left’ and ‘right’ classes were all correctly classified instances, respectively. Misclassifications for these classes were relatively few, with errors mainly distributed across adjacent directional inputs. The ‘select’ class presented relatively low accuracy compared to the other commands, with 504 of 641 instances correctly recognized, and some degree of confusion occurring with the ‘down’, ‘up’, and ‘left’ classes.

The overall average accuracy was 88.2%, indicating that the system maintained practical classification performance under online conditions. Class-specific quantitative metrics recorded an average precision of 87.3%, average recall of 90.6%, and an average F1-score of 88.3%. The ‘up’, ‘left’, and ‘right’ inputs showed consistently high recognition performance. Conversely, the ‘down’ input demonstrated reduced precision of 71.0%, and the ‘select’ input exhibited relatively low recall of 75.8% despite a high precision of 98.4%. These results suggest that some ‘down’ and ‘select’ actions were confused with directional inputs of similar activation patterns. Analysis of the information transfer rate revealed an average of 92.87 bits/min across the three subjects, indicating that despite its simple feature-based algorithm, the proposed system sustained stable and efficient communication in real-world environments.

These results demonstrate that the proposed system maintains stable classification performance overall, even under conditions where users perform multiple actions consecutively as if typing characters. Notably, even when subjects repeatedly performed the same action multiple times, we confirmed that simple feature extraction algorithms based on SSC and RMS alone could effectively distinguish between signals with similar characteristics across classes.

To comprehensively evaluate the input recognition performance and user applicability of the proposed system, the results of Experiment 1 and Experiment 2 were compared. In Experiment 1, conducted with a limited number of inputs and in a controlled environment, the system demonstrated very high classification performance, achieving an overall average accuracy of 90.0%, precision of 95.7%, recall of 90.0%, F1-score of 89.8%, and information transfer rate of 99.5 bits/min. Notably, the inputs ‘up’, ‘left’, and ‘right’ were all perfectly classified, confirming that the system operates with high reliability for basic directional inputs.

In Experiment 2, even as the number of inputs increased and experiments were conducted under more diverse conditions, the overall average accuracy remained at 88.65%, precision at 86.45%, recall at 91.15%, F1-score at 87.8%, and information transfer rate at 92.87 bits/min. This is a positive result demonstrating that the system can reliably maintain practical performance levels even as the usage environment expands.

**Figure 10:**
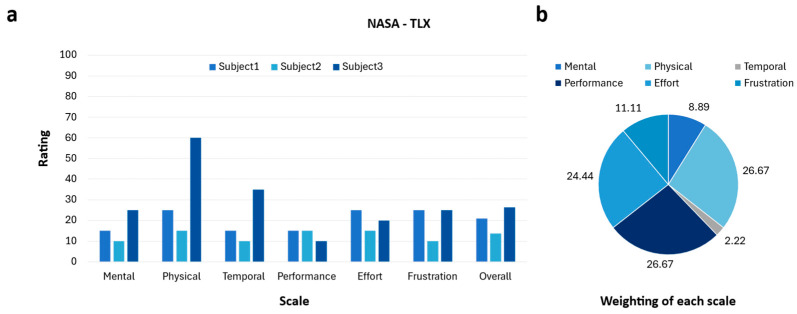
Results of classification performance and user workload assessment; (**a**) Bar graph presenting evaluation scores of six workload components and overall workload across three subjects; (**b**) Pie chart showing the proportional contribution of each component to the overall workload.

Additionally, to assess the subjects’ subjective cognitive workload, the NASA-TLX questionnaire was administered after the experiment concluded. The results are presented in Figure 10. This questionnaire is structured to evaluate six items: Mental Demand, Physical Demand, Temporal Demand, Performance, Effort, and Frustration. Across participants, the overall workload scores ranged from 13.7 to 26.3, corresponding to a medium perceived workload level. As shown in Figure 10a, most individual item scores remained relatively low, generally between 10 and 30 points, with the exception of Physical Demand and Temporal Demand, which reached higher values for certain subjects. The pie chart in Figure 10b illustrates the relative weighting of each item, showing that Physical Demand accounted for the largest proportion at 26.7%, followed by Performance at 26.4% and Effort at 24.4%. Temporal Demand showed the lowest contribution, and both Mental Demand and Frustration were also minor components of the overall workload. These results confirm that while the proposed input system may impose some degree of physical burden, particularly in terms of muscular effort, it does not generate excessive cognitive load, time pressure, or emotional stress. Overall, participants perceived the workload as moderate and manageable, suggesting that the system can be adopted without substantial difficulty.

## 4. Discussion

The proposed EMG-based Cheonjiin speller demonstrated stable and accurate performance across both experimental conditions. The system achieved overall accuracy near 89% and maintained comparable precision and recall values between simple and continuous input tasks, confirming its robustness during real-time operation. The average information transfer rate (ITR) of 96.19 bits/min indicates that the system enables interactive communication while operating with only two EMG channels. This level of performance is comparable to previously reported EMG-based interfaces (40–90 bits/min) [24] and approaches that of mid-level SSVEP-based BCI spellers (80–100 bits/min) [22,23].

These findings suggest that the proposed system can achieve competitive throughput while maintaining low computational cost and real-time responsiveness, as evidenced by the mean command interval of approximately 2.2 s. Moreover, the results reflect consistent user–machine coordination and minimal interaction conflict, underscoring the system’s practical applicability even under low-resource hardware conditions [1].

It demonstrates that stable classification of up/down, left/right, and selection inputs is achievable using only simple time-domain features based on RMS and SSC, without complex deep learning models. The observed results demonstrate that the selected time-domain features effectively capture the physiological characteristics of muscle activation relevant to directional control. In particular, RMS exhibited clear increases corresponding to contraction intensity, indicating its role as a direct measure of muscle activation strength. SSC, in contrast, revealed more dynamic fluctuations, reflecting transient variations in muscle fiber recruitment and firing behavior during directional transitions. These complementary patterns were especially evident between the rectus femoris and gastrocnemius muscles, whose activation dynamics differed across up, down, left, and right commands. Together, RMS and SSC enabled consistent differentiation of movement directions while maintaining low computational complexity. This confirms that the chosen features not only simplify the real-time processing pipeline but also retain sufficient discriminative power for reliable classification in practical EMG-based communication systems.

Compared to recent EMG-based speller systems that utilize multi-channel setups or complex frequency-domain features [21,36], the proposed system demonstrates that comparable accuracy can be achieved with only two channels and simple time-domain descriptors. This finding underscores the potential of the system as a minimal yet effective approach for real-time text input, particularly in low-resource or embedded environments.

While previous studies typically employed deep learning-based models for EMG signal analysis and classification [37,38,39], such approaches often require large datasets, high computational resources, and extended training times, which limit their feasibility for real-time or embedded applications [40]. In contrast, the present study effectively distinguished similar EMG signals using simple time-domain features such as RMS and SSC, demonstrating that reliable classification can be achieved with minimal computational cost. This lightweight feature-based approach aligns with findings from prior research on efficient EMG-based control paradigms [41,42], confirming its suitability for portable assistive systems.

Furthermore, this system provides a practical alternative input method for users with limited hand mobility. By adopting the Cheonjiin layout, which has fewer keys and shorter key travel distances compared to the QWERTY layout, Korean input is possible using only directional control operations. Minimizing input actions is expected to reduce user burden. Electrode placement and input actions were set based on anatomical structures, maintaining high consistency and accuracy despite differences in signal strength and variation patterns between subjects.

Despite the overall high accuracy observed in both experiments, some misclassifications were identified—particularly in the “select” command, which was occasionally recognized as “up” or “left.” This phenomenon is likely attributed to the challenge of isolating specific lower-limb muscles, as some participants unintentionally activated adjacent muscles during contraction. Even when participants intentionally attempted to activate the muscles corresponding to “select,” the signal from one muscle (e.g., the rectus femoris) could be detected earlier or more strongly than its counterpart (e.g., the gastrocnemius), resulting in an unbalanced feature pattern. Additionally, occasional confusion in directional intent was observed when participants rapidly switched between directional and selection commands, indicating a possible timing mismatch between muscle activations and the system’s recognition window.

To mitigate such errors, future implementations could increase the duration of each voluntary contraction or introduce adaptive timing calibration to better align the recognition window with the user’s activation dynamics. Moreover, as users become more accustomed to the directional control scheme through repeated practice, such misclassifications are expected to decrease due to improved motor consistency and proprioceptive control. Therefore, incorporating adaptive calibration and user-specific training sessions may further enhance system reliability.

The present research should be further expanded to broaden its scope and practical applicability. First, the study involved a limited number of healthy participants, which may constrain the generalizability of the results to individuals with motor impairments. Expanding the participant group to include individuals with motor impairments would enhance the generalizability of the findings and provide a more comprehensive evaluation of the system’s clinical applicability. However, recruiting patients with movement disorders within a limited timeframe poses practical challenges, including the need for additional institutional review board (IRB) approval and ethical considerations related to patient safety and consent.

Future studies should investigate how different muscle contraction patterns vary across demographic and physiological factors. For instance, differences between young and middle-aged adults, male and female participants (influenced by muscle mass and fat distribution), and individuals with varying body mass index (BMI) or activity levels could significantly affect EMG amplitude and stability. Comparative analysis among healthy participants, those with mild motor impairments, and those with severe disabilities would provide deeper insights into system adaptability. In addition, systematic evaluation of contraction parameters—such as contraction intensity (e.g., weak 10–20% vs. strong 50–70%), contraction duration (short tap vs. sustained hold), fatigue accumulation across repeated tasks, and sensitivity differences between the rectus femoris and gastrocnemius—would help optimize classifier robustness and improve usability across diverse user groups.

Second, the experimental setup was conducted in a controlled environment, whereas real-world conditions may introduce additional noise or signal variability. Third, while the current study employed static feature parameters, adaptive or context-aware algorithms could further improve robustness under changing physiological states or electrode conditions. Addressing these limitations in future studies will be critical for achieving practical deployment in daily assistive contexts.

Another important consideration for future development is the effect of muscle fatigue on EMG signal stability and classification performance. Since the proposed system relies on voluntary contractions of the rectus femoris and gastrocnemius muscles, prolonged or repeated activation may lead to muscle fatigue, potentially reducing signal amplitude and altering spectral characteristics. This issue is particularly relevant for lower-limb muscles, which may fatigue faster under repetitive activation compared to smaller upper-limb groups. Prior research has demonstrated that fatigue induces a shift in the median frequency of EMG signals, which is closely related to changes in the zero-crossing rate and slope sign change (SSC) features [43]. Because SSC was one of the key features used in our study, such variability could influence the reliability of long-term classification. Therefore, future studies should investigate adaptive feature selection or fatigue-compensation mechanisms to ensure stable performance during extended use sessions.

From a software architecture perspective, the current implementation also presents opportunities for further development. First, the entire signal acquisition, feature extraction (RMS, SSC, and peak amplitude), and classification pipeline was implemented in Python, which—while highly productive and convenient for rapid prototyping—carries inherent overhead. For example, the interpreted nature of Python, the Global Interpreter Lock (GIL), and non-deterministic garbage collection can introduce latency or jitter, especially under strict real-time constraints [44]. Secondly, while our latency (~2.17 s) is acceptable for the targeted interface scenario, it remains relatively coarse compared to hard real-time control systems where latencies of a few milliseconds or hundreds of microseconds are required [45].

In future work, we plan to explore a hybrid architecture in which latency-critical components (e.g., real-time signal preprocessing and event triggering) are implemented in a compiled language such as C or C++ and invoked from Python only for higher-level tasks. This would reduce end-to-end latency and improve determinism under wearable or embedded deployment.

While the system performs well with simple time-domain features, future research could explore advanced feature extraction or hybrid modeling techniques to further improve classification performance. Approaches such as wavelet transform-based time–frequency analysis or combined statistical–spectral features have shown promise in capturing both transient and steady-state components of EMG signals [29,46]. Integrating such hybrid representations with lightweight classifiers could enhance accuracy while maintaining real-time feasibility. This approach facilitates model lightweighting and power consumption reduction, holding significant promise for future applications in portable assistive devices or low-specification environments.

Future technical refinements hold potential to further enhance the system’s practicality and accessibility. For instance, integrating adaptive filtering algorithms capable of real-time noise estimation [47], or implementing calibration-free signal normalization schemes [48], could improve robustness across sessions. Hardware improvements, such as wireless sensor integration and flexible electrode materials [49], may also increase portability and user comfort. Furthermore, by utilizing diverse sensory channels like vision, hearing, and touch to enable users to intuitively perceive the system’s responses, it is anticipated that the system could be expanded into a comprehensive Augmentative and Alternative Communication (AAC) system accessible to users with visual or auditory impairments.

Ultimately, our study demonstrates the potential for electromyography (EMG)-based interaction technology to substantially enhance information accessibility and freedom of expression for users with physical limitations. It can serve as foundational data for future user-centered interface design.

## 5. Conclusions

Our study introduced a lightweight EMG-based Cheonjiin speller system capable of Korean text input through five directional muscle commands. The system achieved an overall accuracy of 88.9% and an information transfer rate of 96.19 bits/min, demonstrating its feasibility for real-time communication.

The key contribution of this work lies in showing that accurate EMG-based interaction can be achieved using simple time-domain features—RMS and SSC—without relying on complex machine-learning models. By integrating the directional input structure with the Cheonjiin layout, the system provides an intuitive, low-cost, and accessible communication method for users with physical impairments.

Beyond text input, the proposed framework has broader implications for the field of human–machine interaction. Potential application areas include hands-free device control in smart environments, gaming and virtual-reality interaction, rehabilitation and motor recovery monitoring [50], silent communication interfaces in noisy or privacy-sensitive contexts, and wearable bio-signal systems for continuous healthcare and Internet of Things (IoT) applications.

In summary, this research establishes a practical foundation for lightweight, portable EMG-based assistive communication systems that enhance accessibility, communication freedom, and user independence, while contributing to the growing integration of physiological signals in next-generation interactive technologies.

## Figures and Tables

**Table 1:** Composition rules and resulting consonant characters in the Cheonjiin keyboard layout.

Category	Base Key(s)	Composition Rule	Resulting Characters
Consonant	ㄱㅋ	Single: ㄱ; Double: ㅋ; Triple: ㄲ	ㄱ, ㅋ, ㄲ
	ㄴㄹ	Single: ㄴ; Double: ㄹ;	ㄴ, ㄹ
	ㄷㅌ	Single: ㄷ; Double: ㅌ; Triple: ㄸ	ㄷ, ㅌ, ㄸ
	ㅂㅍ	Single: ㅂ; Double: ㅍ; Triple: ㅃ	ㅂ, ㅍ, ㅃ
	ㅅㅎ	Single: ㅅ; Double: ㅎ; Triple: ㅆ	ㅅ, ㅎ, ㅆ
	ㅈㅊ	Single: ㅈ; Double: ㅊ; Triple: ㅉ	ㅈ, ㅊ, ㅉ
	ㅇㅁ	Single: ㅇ; Double: ㅁ	ㅇ, ㅁ
Vowel	ㅣ		ㅣ
	ㆍ		ㆍ
	ㅡ		ㅡ
	ㅣ + ㆍ		ㅏ
	ㆍ + ㅣ		ㅓ
	ㅣ + ㆍ + ㆍ		ㅑ
	ㆍ + ㆍ + ㅣ		ㅕ
	ㆍ + ㅡ		ㅗ
	ㅡ + ㆍ		ㅜ
	ㆍ + ㆍ + ㅡ		ㅛ
	ㅡ + ㆍ + ㆍ		ㅠ
	ㅡ + ㅣ		ㅢ
	ㆍ + ㅡ + ㅣ		ㅚ
	ㅡ + ㆍ + ㅣ		ㅟ
	ㅣ + ㆍ + ㅣ		ㅐ
	ㆍ + ㅣ + ㅣ		ㅔ
	ㅣ + ㆍ + ㆍ + ㅣ		ㅒ
	ㆍ + ㆍ + ㅣ + ㅣ		ㅖ
	ㆍ + ㅡ + ㅣ + ㆍ+ ㅣ		ㅙ
	ㆍ + ㅡ + ㆍ + ㅣ + ㅣ		ㅞ
	ㆍ + ㅡ + ㅣ + ㆍ + ㅣ		ㅝ

## Data Availability

The original contributions presented in the study are included in the article, further inquiries can be directed to the corresponding authors.

## References

[1] Pan Y., Xu J. (2025). Human–machine plan conflict and conflict resolution in a visual search task. Int. J. Hum.–Comput. Stud..

[2] Cai L., Yan S., Ouyang C., Zhang T., Zhu J., Chen L., Ma X., Liu H. (2023). Muscle synergies in joystick manipulation. Front. Physiol..

[3] Bizzi E., Cheung V.C. (2013). The neural origin of muscle synergies. Front. Comput. Neurosci..

[4] Clark D.J., Ting L.H., Zajac F.E., Neptune R.R., Kautz S.A. (2010). Merging of healthy motor modules predicts reduced locomotor performance and muscle coordination complexity post-stroke. J. Neurophysiol..

[5] Coscia M., Cheung V.C., Tropea P., Koenig A., Monaco V., Bennis C., Micera S., Bonato P. (2014). The effect of arm weight support on upper limb muscle synergies during reaching movements. J. Neuroeng. Rehabil..

[6] d’Avella A., Saltiel P., Bizzi E. (2003). Combinations of muscle synergies in the construction of a natural motor behavior. Nat. Neurosci..

[7] Hoc J.-M. (2000). From human–machine interaction to human–machine cooperation. Ergonomics.

[8] Inga J., Ruess M., Robens J.H., Nelius T., Rothfuß S., Kille S., Dahlinger P., Lindenmann A., Thomaschke R., Neumann G. (2023). Human–machine symbiosis: A multivariate perspective for physically coupled human–machine systems. Int. J. Hum.–Comput. Stud..

[9] Itoh M., Flemisch F., Abbink D. (2016). A hierarchical framework to analyze shared control conflicts between human and machine. IFAC-Pap..

[10] Chandramouli C., Agarwal V. Speech recognition based computer keyboard replacement for the quadriplegics, paraplegics, paralytics and amputees. Proceedings of the 2009 IEEE International Workshop on Medical Measurements and Applications.

[11] Hayashi H., Tsuji T. (2022). Human–machine interfaces based on bioelectric signals: A narrative review with a novel system proposal. IEEJ Trans. Electr. Electron. Eng..

[12] Li Y., Zhang H. (2025). EEG Signal Recognition of VR Education Game Players Based on Hybrid Improved Wavelet Threshold and LSTM. Int. Arab J. Inf. Technol..

[13] Henderson J.M., Shinkareva S.V., Wang J., Luke S.G., Olejarczyk J. (2013). Predicting cognitive state from eye movements. PLoS ONE.

[14] Jiang H., Wang Z., Jiao R., Jiang S. (2020). Picture-induced EEG signal classification based on CVC emotion recognition system. Comput. Mater. Contin..

[15] López A., Ferrero F., Villar J.R., Postolache O. (2020). High-performance analog front-end (AFE) for EOG systems. Electronics.

[16] Attar E.T. (2022). Review of electroencephalography signals approaches for mental stress assessment. Neurosci. J..

[17] Al-Ayyad M., Owida H.A., De Fazio R., Al-Naami B., Visconti P. (2023). Electromyography monitoring systems in rehabilitation: A review of clinical applications, wearable devices and signal acquisition methodologies. Electronics.

[18] Kwon Y.D., Shatilov K.A., Lee L.-H., Kumyol S., Lam K.-Y., Yau Y.-P., Hui P. Myokey: Surface electromyography and inertial motion sensing-based text entry in AR. Proceedings of the 2020 IEEE International Conference on Pervasive Computing and Communications Workshops (PerCom Workshops).

[19] Wang Y., Wang Y., Zhao R., Shi Y., Bian Y. Efficient electromyography-based typing system: Towards a novel approach to HCI text input. Proceedings of the 2024 46th Annual International Conference of the IEEE Engineering in Medicine and Biology Society (EMBC).

[20] Prasad P.S., Swarnkar R., Hashmi M.F., Keskar A.G. (2017). Design and Implementation of a speller based on EMG signal. Int. J. Comput. Intell. Syst..

[21] Lin K., Chen X., Huang X., Ding Q., Gao X. A hybrid BCI speller based on the combination of EMG envelopes and SSVEP. Proceedings of the Applied Informatics.

[22] López L.I.B., Ferri F.M., Zea J., Caraguay Á.L.V., Benalcázar M.E. (2024). CNN–LSTM and post-processing for EMG-based hand gesture recognition. Intell. Syst. Appl..

[23] Jabbari M., Khushaba R.N., Nazarpour K. EMG-based hand gesture classification with long short-term memory deep recurrent neural networks. Proceedings of the 2020 42nd Annual International Conference of the IEEE Engineering in Medicine & Biology Society (EMBC).

[24] Atzori M., Cognolato M., Müller H. (2016). Deep learning with convolutional neural networks applied to electromyography data: A resource for the classification of movements for prosthetic hands. Front. Neurorobot..

[25] SangHoon L. (2009). 이동통신 단말기의 문자입력방법 및 이를 적용한 이동통신 단말기. KR Patent.

[26] OpenBCI Ganglion Board Technical Specifications. https://docs.openbci.com/Ganglion/GanglionSpecs/.

[27] Microchip Technology Inc. (2020). MCP3912-3V Four-Channel Analog Front End, DS20005348C.

[28] Ou J., Li N., He H., He J., Zhang L., Jiang N. (2024). Detecting muscle fatigue among community-dwelling senior adults with shape features of the probability density function of sEMG. J. Neuroeng. Rehabil..

[29] Phinyomark A., Khushaba R.N., Scheme E. (2018). Feature extraction and selection for myoelectric control based on wearable EMG sensors. Sensors.

[30] Hudgins B., Parker P., Scott R.N. (2002). A new strategy for multifunction myoelectric control. IEEE Trans. Biomed. Eng..

[31] Zhou S., Yin K., Fei F., Zhang K. (2019). Surface electromyography–based hand movement recognition using the Gaussian mixture model, multilayer perceptron, and AdaBoost method. Int. J. Distrib. Sens. Netw..

[32] Li N., Ou J., He H., He J., Zhang L., Peng Z., Zhong J., Jiang N. (2024). Exploration of a machine learning approach for diagnosing sarcopenia among Chinese community-dwelling older adults using sEMG-based data. J. Neuroeng. Rehabil..

[33] Wang B., Sun J., Peng B., Cui X., Cheng L., Zheng X. (2025). Optimal event-triggered neural learning tracking control for pneumatic muscle antagonistic joint with asymmetric constraints. IEEE Trans. Ind. Electron..

[34] Categorical A. (1998). Glossary of terms. Mach. Learn..

[35] Powers D.M. (2020). Evaluation: From precision, recall and F-measure to ROC, informedness, markedness and correlation. arXiv.

[36] Rezeika A., Benda M., Stawicki P., Gembler F., Saboor A., Volosyak I. 30-targets hybrid BNCI speller based on SSVEP and EMG. Proceedings of the 2018 IEEE International Conference on Systems, Man, and Cybernetics (SMC).

[37] Scheme E., Englehart K. (2011). Electromyogram pattern recognition for control of powered upper-limb prostheses: State of the art and challenges for clinical use. J. Rehabil. Res. Dev..

[38] Côté-Allard U., Fall C.L., Drouin A., Campeau-Lecours A., Gosselin C., Glette K., Laviolette F., Gosselin B. (2019). Deep learning for electromyographic hand gesture signal classification using transfer learning. IEEE Trans. Neural Syst. Rehabil. Eng..

[39] Buchman D., Drozdov M., Krilavičius T., Maskeliūnas R., Damaševičius R. (2022). Pedestrian and animal recognition using Doppler radar signature and deep learning. Sensors.

[40] Farina D., Aszmann O. (2014). Bionic limbs: Clinical reality and academic promises. Sci. Transl. Med..

[41] Oskoei M.A., Hu H. (2008). Support vector machine-based classification scheme for myoelectric control applied to upper limb. IEEE Trans. Biomed. Eng..

[42] Sharma R. (2023). Automated human emotion recognition using hybrid approach based on sensitivity analysis on residual time–frequency plane with online learning algorithm. Biomed. Signal Process. Control.

[43] Kim H., Lee J., Kim J. (2018). Electromyography-signal-based muscle fatigue assessment for knee rehabilitation monitoring systems. Biomed. Eng. Lett..

[44] Cho S.Y., Delgado R., Choi B.W. (2023). Feasibility study for a python-based embedded real-time control system. Electronics.

[45] Adam G.K. (2021). Real-time performance and response latency measurements of Linux kernels on single-board computers. Computers.

[46] Karheily S., Moukadem A., Courbot J.-B., Abdeslam D.O. (2022). sEMG time–frequency features for hand movements classification. Expert Syst. Appl..

[47] Boyer M., Bouyer L., Roy J.-S., Campeau-Lecours A. (2023). Reducing noise, artifacts and interference in single-channel EMG signals: A review. Sensors.

[48] Halaki M., Ginn K. (2012). Normalization of EMG signals: To normalize or. Computational Intelligence in Electromyography Analysis: A Perspective on Current Applications and Future Challenges.

[49] Cheng L., Li J., Guo A., Zhang J. (2023). Recent advances in flexible noninvasive electrodes for surface electromyography acquisition. NPJ Flex. Electron..

[50] Kou J., Wang Y., Chen Z., Shi Y., Guo Q., Xu M. (2024). Flexible assistance strategy of lower limb rehabilitation exoskeleton based on admittance model. Sci. China Technol. Sci..

